# Retrospective Analysis of the Effects of the COVID-19 Pandemic on the Emergency Department Walk-Out Rate in an Acute Care Community Hospital During the USA National Lockdown

**DOI:** 10.7759/cureus.16660

**Published:** 2021-07-27

**Authors:** Michael Yakobi, David Cheng MD

**Affiliations:** 1 Emergency Department, Thomas Jefferson University Hospital, Philadelphia, USA; 2 Emergency Department, Akron General Hospital (AGH) at Wright State University (WSU), Akron, USA

**Keywords:** emergency department, covid - 19 pandemic, walk out rates, acute care community hospitals, national lockdown

## Abstract

Background

There are currently no data available that compares the volume and the walk-out rate of patients who presented to the emergency department (ED) before and after the coronavirus disease 2019 (COVID-19) peak pandemic period during the USA national lockdown.

Objective

EDs measure metrics such as walk-out rate (WOR), which is reflected in leaving against medical advice (AMA), elopement, left after triage (LAFT), and left without being seen (LWBS). In this study, we sought to determine the effects of the COVID-19 pandemic on the ED WOR.

Methods

A retrospective analysis was used to assess the overall ED census and the walk-out rate for three community EDs during the March to June 2020 national lockdown. This period was compared to the same period for 2019.

Results

The walk-out rate during the COVID-19 pandemic was inversely proportional to the overall ED census. While overall ED visits decreased by 26% to 42% during the period of March to June 2020 as compared to March to June 2019, the patient walk-out rates increased from 25% to 65%.

Conclusion

The national lockdown during the COVID-19 pandemic created a paradoxical finding with a decrease in ED census but an increase in ED walk-out rates. This decline in census and increase in WOR raises concern that patients avoid going to the hospitals to seek medical attention because the concern of contracting the virus prevails, resulting in increased walkouts. With this retrospective study, we can anticipate that future newly discovered emerging diseases causing national lockdowns will result in a high probability of avoidance of emergency care.

## Introduction

Emergency departments (ED) see a variety of pathologies ranging from non-urgent to emergent conditions. From March to June 2020, during the peak of the coronavirus disease 2019 (COVID-19) pandemic, EDs experienced a decrease in overall census both in the US as well as in other countries [1-2]. This was largely attributed to the patients' concern about contracting the virus and a preference to treat and monitor symptoms at home [2]. A retrospective study was conducted to determine if other metrics, specifically those that measure patient walk-outs, including patients who left without being seen (LWBS), patients who left prior to triage (LPT), patients who left after triage (LAFT), patients who left against medical advice (AMA), and patient elopement, were affected as well. The study emphasizes that while the overall patient census may decrease amid concerns about contracting the virus, providers must reinforce the importance of timely care for medical emergencies.

## Materials and methods

A retrospective analysis assessed the monthly overall ED census and the walk-out rate (WOR) for three EDs that are geographically distant from March to June 2020. This period was compared to the same period in 2019. To reiterate, the walk-out rate was defined as the summation of the following metrics: patients who left without being seen (LWBS), patients who left prior to triage (LPT), patients who left after triage (LAFT), patients who left against medical advice (AMA), and patient elopement.

Data analysis was conducted using the administrative dashboard of metrics found in the electronic medical record of three hospitals that are in the same network. The hospitals included in this study are located in Northern New Jersey, Bucks County, Pennsylvania, and Dallas, Texas (Hospitals A, B, and C, respectively). They are 120, 170, and 200-bed capacity hospitals, respectively. All hospitals are acute care community hospitals in urban settings.

The data were collected electronically by the EPIC electronic medical system. Specifically, upon completion of medical workup, a patient disposition is reported. These dispositions are reported as either admitted or discharged if a workup is complete. However, if no disposition is reached because the patient refused to complete the workup or walked out prior to the evaluation, the disposition is reported as LWBS, LPT, LAFT, or AMA. These metrics appear numerically on the dashboard along with census metrics.

No patient information or identifiers or any kind were provided in the data sets provided. All data analyzed were strictly limited to hospital metrics regarding hospital census and walk-out rates. Due to the proprietary nature of the information, only the hospitals’ location and its bed capacity were included.

There was no financial conflict of interest in this review.

## Results

For each of the three hospitals, the WORs during the COVID-19 pandemic were inversely proportional to the overall ED census.

Hospital A, a 120-bed community hospital in Northern NJ, had an ED census decline of 42% while WORs increased by 25% (Table 1).

**Table 1:** Hospital A: Northern NJ ED: emergency department; LWBS: patients who left without being seen; LPT: patients who left prior to triage; LAFT: patients who left after triage; AMA: patients who left against medical advice

Hospital A	TOTAL Census 2019	TOTAL Census 2020	∆										
ED Visits	16600	9647											
LWBS	84	83											
%LWBS	0.51%	0.86%	70%										
LPT	3	10											
%LPT	0.02%	0.10%	474%										
LAFT	81	73											
%LAFT	0.49%	0.76%	55%										
AMA	330	202											
%AMA	1.99%	2.09%	5%										
Elope	226	157											
%Elope	1.36%	1.63%	20%										
TOTAL WALK-OUTS	4.36%	5.42%	25%										
				The census at Hospital A in 2019 March - June was 16,640 and 9647 during the same period in 2020.									

Hospital B, a 170-bed community hospital in Bucks County, PA, had an ED census decline of 38% while patient WORs increased by 65% (Table 2).

**Table 2:** Hospital B: Bucks County, Pennsylvania ED: emergency department; LWBS: patients who left without being seen; LPT: patients who left prior to triage; LAFT: patients who left after triage; AMA: patients who left against medical advice

HOSPITAL B	TOTAL Census 2019	TOTAL Census 2020	∆										
ED Visits	8252	5124											
LWBS	42	56											
%LWBS	0.51%	1.09%	115%										
LPT	16	22											
%LPT	0.19%	0.43%	121%										
LAFT	26	34											
%LAFT	0.32%	0.66%	111%										
AMA	115	87											
%AMA	1.39%	1.70%	22%										
Elope	58	64											
%Elope	0.70%	1.25%	78%										
TOTAL WALK-OUTS	3.11%	5.13%	65%	The census at Hospital B in 2019 March - June was 8252 and 5124 during the same period in 2020									

Finally, Hospital C, a 200-bed community hospital in Dallas, TX, had an ED census decline of 26% while patient WORs increased by 64% (Table 3).

**Table 3:** Hospital C: Dallas, Texas ED: emergency department; LWBS: patients who left without being seen; LPT: patients who left prior to triage; LAFT: patients who left after triage; AMA: patients who left against medical advice

HOSPITAL C	TOTAL Census 2019	TOTAL Census 2020	∆										
ED Visits	13862	10305											
LWBS	230	291											
%LWBS	1.66%	2.82%	70%										
LPT	74	83											
%LPT	0.54%	0.81%	51%										
LAFT	156	208											
%LAFT	1.13%	2.02%	79%										
AMA	147	148											
%AMA	1.06%	1.44%	35%										
Elope	95	126											
%Elope	0.69%	1.23%	78%										
TOTAL WALK-OUTS	5.06%	8.31%	64%	The census at Hospital C in 2019 March - June was 13862 and 10305 during the same period in 2020.									

## Discussion

One of the consequences of the COVID-19 pandemic was a decrease in the overall ED census, a phenomenon observed both in the US as well as other countries [3-4]. We hypothesize that this trend was largely attributed to the patients' concern about contracting the virus in hospital settings, as well as a preference to treat and monitor symptoms at home [5]. Because of these concerns, patients delayed seeking medical care for symptoms such as chest pain or neurological deficit, suggesting patients were not seeking necessary care in a timely manner [6]. Overall, COVID-19-suspected cases rapidly increased in the ED while non-COVID-19 suspected cases declined and remained well below those of the pre-COVID-19 era [7].

In our study, we observed the same decline in the overall ED census but an unexpected increase in WOR during the peak of the COVID-19 pandemic from March to June 2020. Because there were no other changes in the ED, such as medical staff reduction, major delays in diagnostic tests, or boarding in the ED, patient walkouts were likely due to the fear of virus exposure while in the ED [8].

Specifically, this study showed that the COVID-19 pandemic lockdown had many effects on healthcare systems, ranging from decreased ED census, increased incidence of patients canceling outpatient clinic visits, increased incidence of postponed elective procedures and surgeries, and even a delay in care of ST-elevation myocardial infarctions [9-11]. In addition, a study indicated that both the census and triage acuity declined significantly [12]. This trend again raises the concern that sick patients were not seeking medical attention [12]. The fear of contracting COVID-19 if one went to the hospital seemed to be the most cited reason [13]. Recently, a pilot study has created a COVID-19 Phobia Scale (C19P-S) [14]. The scale is calculated from a questionnaire administered to patients regarding psychological, psychosomatic, economic, and social factors. In our retrospective analysis of available data, this concern of a COVID-19 phobia not only prevented patients from seeking medical attention, resulting in the decrease in the overall census, but once in the ED, the heightened concern of contracting the virus became more prevalent as they became surrounded by the number of COVID-19 patients treated in the ED, resulting in increased walk-outs. Overall, the decreasing census, increasing delays in seeking medical care, and increasing patient walk-outs have significant risks for patients’ health. Knowing this trend exists, both providers and administrators must do their best to assure patients that the hospital is taking all the measures to create a safe environment and isolate patients from the potential hazard of contracting the infection as well as encourage the patients to seek medical care for their health problems. In practice, this would include advertisements that increase transparency regarding the sanitation processes the hospital has employed. It may also include having pamphlets available, which include the Center for Disease Control's (CDC's) published data on virus transmission rates and interventions the hospital is using (hand sanitizer stations, mask/N95 use, staff vaccination rates).

The limitations of this study include being retrospective in nature, using descriptive statistics, a community hospital setting, and a brief national lockdown time period. Perhaps the most significant limitation to this study includes a slight overestimation of walk-out rates directly related to the COVID-19 pandemic. This study infers that all patients who left EDs did so for reasons related to the COVID-19 pandemic. In reality, patients who walk out from EDs do not cite a reason. In the pre-pandemic period, typical walk-out reasons included child or elderly care, and an inability to miss work or important socials events. However, during the pandemic, most stayed home and social events were canceled. During the peak of the COVID-19 pandemic, the vast majority of patients who did leave likely did so for reasons related to the pandemic with a preference to treat and monitor symptoms at home.

## Conclusions

Despite a decrease in ED census, our study shows a paradoxical increase in WOR. This decline in census and increase in WORs raises concern that patients were concerned enough to go to the emergency department to seek medical attention, however, they left prior to the conclusion of medical assessment and treatment due to concern of contracting the virus. With this retrospective study, we can anticipate that future, newly discovered emerging diseases causing national lockdowns will result in a high probability of nonparticipation in emergency care. Knowing that this trend exists, both providers and administrators must do their best to assure patients that the hospital takes all the measures necessary to assure a safe environment that isolates patients from the potential hazard of contracting infections.
